# Delayed Union of a Diaphyseal Forearm Fracture Associated With Impaired Osteogenic Differentiation of Prospectively Isolated Human Skeletal Stem Cells

**DOI:** 10.1002/jbm4.10398

**Published:** 2020-08-31

**Authors:** L Henry Goodnough, Thomas H Ambrosi, Holly Steininger, Malcolm R DeBaun, Geoffrey D Abrams, Timothy R McAdams, Michael J Gardner, Charles KF Chan, Julius A Bishop

**Affiliations:** ^1^ Department of Orthopaedic Surgery Stanford University School of Medicine Stanford CA USA; ^2^ Institute for Stem Cell Biology and Regenerative Medicine Stanford University School of Medicine Stanford CA USA; ^3^ Department of Surgery Stanford Hospitals and Clinics Stanford CA USA

**Keywords:** BONE CELLS, INJURY/FRACTURE HEALING, ORTHOPEDICS, STROMAL/STEM CELLS

## Abstract

Delayed union or nonunion are relatively rare complications after fracture surgery, but when they do occur, they can result in substantial morbidity for the patient. In many cases, the etiology of impaired fracture healing is uncertain and attempts to determine the molecular basis for delayed union and nonunion formation have been limited. Prospectively isolating skeletal stem cells (SSCs) from fracture tissue samples at the time of surgical intervention represent a feasible methodology to determine a patient's biologic risk for compromised fracture healing. This report details a case in which functional in vitro readouts of SSCs derived from human fracture tissue at time of injury predicted a poor fracture healing outcome. This case suggests that it may be feasible to stratify a patient's fracture healing capacity and predict compromised fracture healing by prospectively isolating and analyzing SSCs during the index fracture surgery. © 2020 The Authors. *JBMR Plus* published by Wiley Periodicals LLC on behalf of American Society for Bone and Mineral Research.

## Introduction

Nonunions of surgically treated forearm fractures are rare, occurring at a rate of 2% to 4%.^(^ ^1^, ^2^ ^)^ Nevertheless, this impaired fracture healing is both disabling to the patient and costly to the health care system.^(^ ^3^, ^4^ ^)^ Although certain injury patterns, patient factors, or technical aspects of surgery are associated with compromised fracture healing,^(^ ^5^, ^6^ ^)^ the etiology of most instances of delayed union or nonunion is often unclear. Although conventionally available laboratory tests can assess a patient's bone mineral homeostasis,^(^ ^7^ ^)^there is currently no reliable way to assess or predict a patient's biologic fracture‐healing capacity.

**Fig 1:**
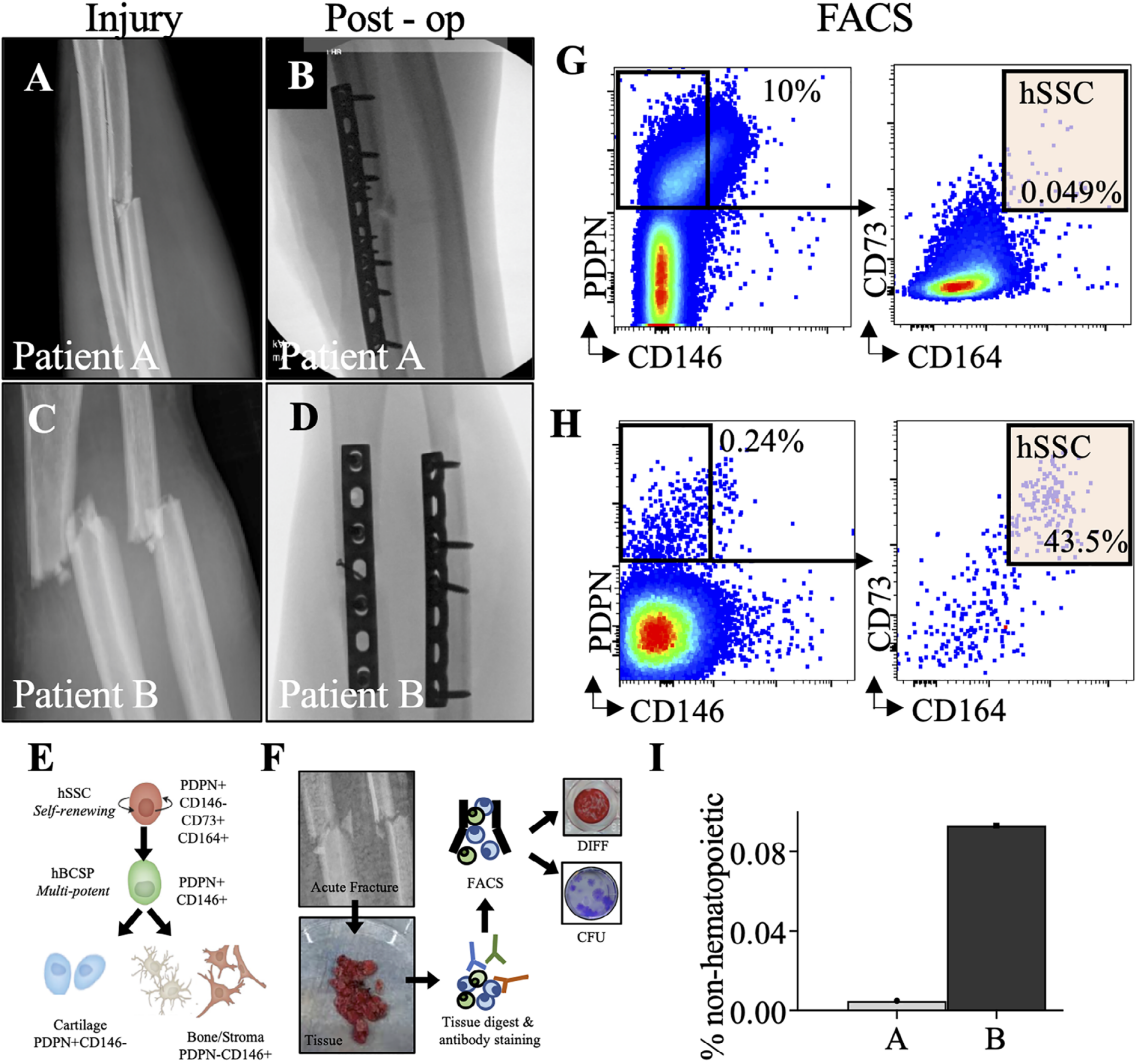
(*A*,*C*) forearm radiographs demonstrating bending wedge diaphyseal ulna fracture at injury and immediately postoperatively. (*B*,*D*) fForearm radiographs demonstrating simple fracture patterns of diaphyseal radius and ulna at injury and immediately post‐operatively. (*E*) Schematic of the human SSC lineage tree with a self‐renewing stem cell at the top of the hierarchy giving rise to a human bone/cartilage/stromal cell progenitor (hBCSP) and to lineage committed cartilage and bone/stromal cells (PDPN: Podoplanin). (*F*) Experimental workflow for SSC isolation from patient tissue. (*G*,*H*) FACS plots demonstrating isolation of SSCs from fractures immediately after surgery. (*I*) Frequency of hSSCS at fracture sites as percentage of non‐hematopoietic cells for normalization.

Impaired fracture healing is thought to originate, at least in part, at the stem cell level.^(^ ^8^, ^9^, ^10^ ^)^ To date, however, efforts to identify stem cell defects have been limited by retrospective study design and the heterogeneous nature of progenitor cell populations.^(^ ^11^, ^12^, ^13^, ^14^, ^15^, ^16^, ^17^ ^)^ Recently, we have described a method of isolating a human skeletal stem cell (hSSC; Fig. 1*A*) using fluorescence‐activated cell sorting (FACS) that is capable of multilineage skeletal differentiation,^(18^ ^)^ allowing us to perform detailed molecular and functional investigations into skeletal development, growth, and regeneration.^(^ ^19^, ^20^ ^)^ In this case report, we describe a situation in which prospectively isolated hSSCs, collected at the time of fracture fixation, predicted compromised fracture healing, despite the fact that the patient was young and healthy, in a fracture at generally low risk for compromised healing and with technically appropriate surgery.

## Methods

This study was approved by the institutional research board stanford university school of medicine. We compared two patients with similar age, sex, and diaphyseal forearm fracture patterns. Both patients underwent surgery between 1 and 3 days from injury. At the time of fracture fixation, hematoma that impeded visualization and/or fracture reduction was removed using a pituitary rongeur and saved for analysis (Fig. 1*B*,*C*).

Skeletal stem cells were purified by FACS from the hematoma of the surgical site and cultured for fibroblastic colony‐forming units (CFU‐F) and osteogenic differentiation as described.^(^ ^18^ ^)^Tissue was isolated from hematoma interposed between fracture fragments at the time of surgery. Samples were first placed on ice and then isolated by FACS. Briefly, tissue was enzymatically digested with 3000 U/mL type II collagenase (Catalog #C6885; Sigma‐Aldrich, St. Louis, MO, USA) digestion buffer supplemented with 100 U/mL DNase I (Catalog #NC9199796; Worthington Biochemical Corporation, Lakewood, NJ, USA) and incubated at 37°C for 60 minutes under constant agitation. After centrifugation, rinsing, and resuspension, cells were stained with fluorochrome‐conjugated antibodies against CD45 (Catalog #304029‐BL; BioLegend, San Diego, CA, USA), CD235a (Catalog #306612‐BL; BioLegend), CD31 (Catalog #13‐0319‐82; Thermo Fisher Scientific, Waltham, MA, USA), CD202b (Tie‐2) (Catalog #334204; BioLegend), CD146 (Catalog #342010; BioLegend), PDPN (Catalog #17‐9381‐42; Thermo Fisher Scientific), CD90 (THY1; Catalog #328110; BioLegend), CD164 (Catalog #324808; BioLegend), and CD73 (Catalog #344016; BioLegend; Fig. 1*D*). Flow cytometry was performed on a FACS Aria II (BD Biosciences, San Jose, CA, USA; Fig. 1*E*). Gating schemes were established with fluorescence‐minus‐one (FMO: staining with all fluorophores except one) controls; negative propidium iodide (Catalog #P4170; Sigma‐Aldrich) staining (1 mg/mL) was used as a measure for cell viability.

SSCs were cultured and assessed for clonogenicity using a CFU‐F assay and for osteogenic differentiation using Alizarin Red staining. Briefly, FACS‐purified hSSCs were cultured in α modified essential medium (α‐MEM) with 10% human platelet‐derived lysate (Catalog #06960; STEMCELL Technologies, Vancouver, Canada), 1% penicillin–streptomycin solution (Pen‐Strep; Catalog #15140‐122; Thermo Fisher Scientific) at 37°C with 5% CO_2_. For CFUs‐F, hSSCs seeded at clonal density were cultured for 10 to 14 days, fixed with formalin, and stained with crystal violet stain (Catalog #C0775; Sigma Aldrich) for counting (Fig. 1*F*).

For osteogenic differentiation, freshly sorted and expanded cells grown to 80% to 90% confluency were supplemented with osteogenic factors to their culture medium containing α‐MEM (Catalog #12561‐056; Thermo Fisher Scientific), 10% FBS, 1% Pen‐Strep, 100nM dexamethasone (Catalog #194561; MP Biomedicals, Santa Ana, CA, USA), 10mM sodium b‐glycerophosphate (Catalog #G9891; Sigma‐Aldrich), 2.5mM ascorbic acid 2‐phosphate (Catalog #A8960; Sigma‐Aldrich). Media were changed every other day, and cells were fixed with 4% formalin at day 14 of differentiation culture for staining with Alizarin Red S (Catalog #A5533; Sigma‐Aldrich; Fig. 1*F*). Each patient was analyzed in triplicate for differentiation. Differentiation was quantified by spectrophotometry using absorbance at 450 nm.

Fracture fixation was performed by a surgeon with subspecialty fellowship training in orthopedic trauma surgery based on well‐described principles of operative fracture management.^(^ ^21^ ^)^

## Results

The index patient (patient A) is a 27‐year‐old, nonsmoking, healthy man who sustained an isolated closed diaphyseal ulna fracture during a sports competition (Fig. 1*A*). His fracture was treated with open reduction and internal fixation with bridge plating (Fig. 1*B*).

Patient B is a 23‐year‐old, nonsmoking, healthy man who sustained an open diaphyseal both bone forearm fracture in a bike accident (Fig. 1*C*). The day of injury, he underwent surgery with lag screw and neutralization plating of his radius and compression plating of his ulna fracture (Fig. 1*D*).

**Fig 2:**
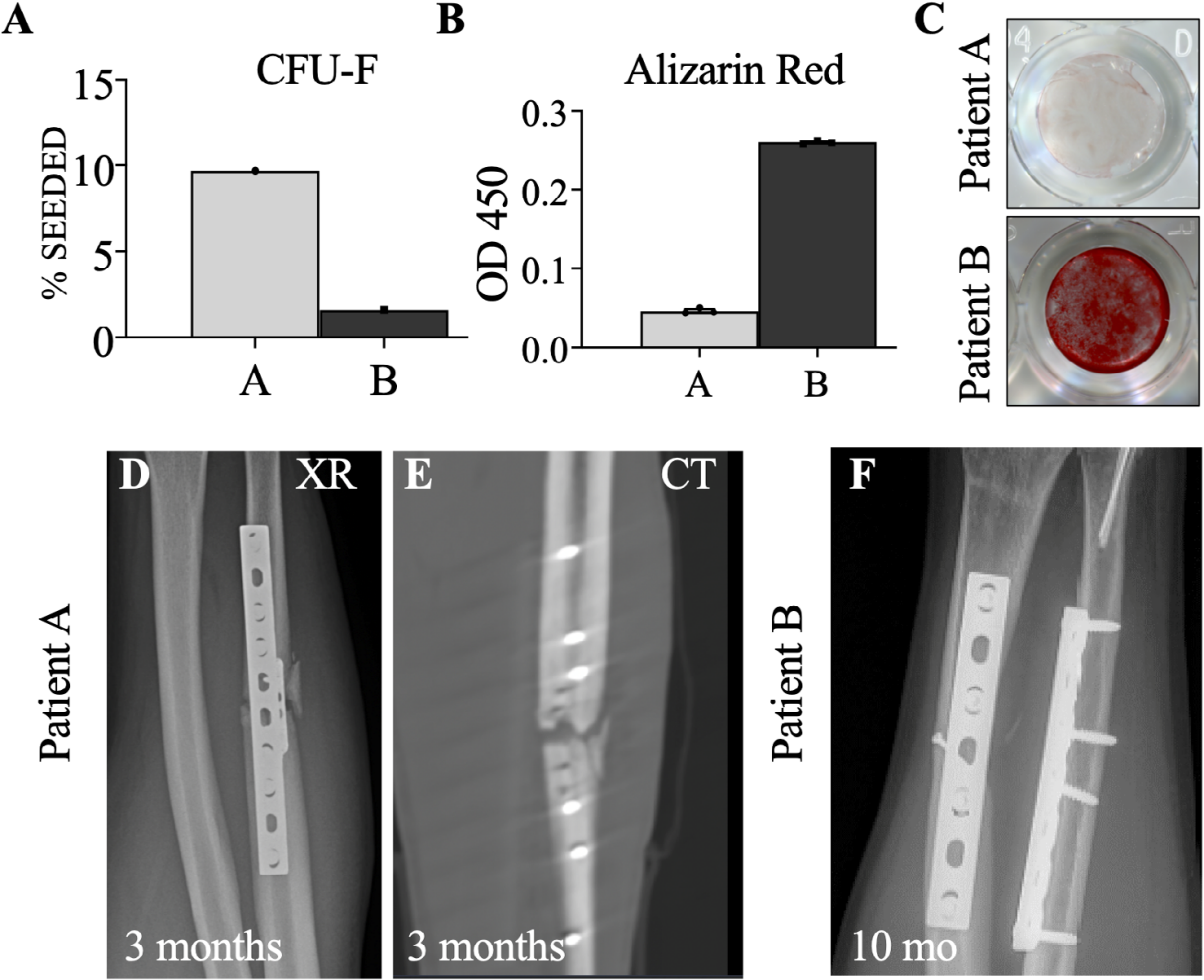
(*A*) Comparison of colony forming unit‐fibroblast (CFU‐F) assay between patients A and B as a percent of hSSCs cultured. (*B*) Alizarin red stain quantification between samples A and B run in triplicate using spectrophotometry with optical density measurement at 450 nm. (*C*) Qualitative comparison of alizarin red staining of hSSCs after osteogenic differentiation. (*D*,*E*) plain radiographs (XR) and computed tomography (CT) at 3months demonstrating absence of callus formation in patient A (*F*). Ten month radiographs of patient B demonstrating healed fractures.

hSSCs were purified from both patients by FACS and cultured (Fig. 1*E*,*F*). Patient B had a slightly greater yield of SSCs (CD45^−^CD235a^−^CD31^−^Tie2^−^CD146^−^PDPN^+^CD73^+^CD164^+^) purified from the fracture site as a proportion of nonhematopoietic nucleated cells (Fig. 2*G* to *I*). hSSCs from both patients yielded CFUs‐F (Fig. 2*A*), with greater frequency in patient A. However, the hSSCs from patient A demonstrated a qualitative and quantitative loss of osteogenic capacity as measured by Alizarin Red staining for mineralization after differentiation (Fig. 2*B*,*C*).

Three months postsurgery, patient A had no evidence of bony healing on radiographs or CT and was diagnosed with a delayed union (Fig. 2*D*,*E*). He was treated with revision of his fracture fixation, as well as supplementation with an iliac crest autograft. Patient B experienced routine fracture healing, with resolution of symptoms and radiographic healing 10 months postsurgery (Fig. 2*F*).

## Discussion

In most patients with delayed fracture healing, the etiology cannot be determined. We describe here a case report of a young, healthy individual who sustained a diaphyseal forearm fracture, in which prospectively isolated SSCs obtained during fracture fixation demonstrated impaired osteogenic capacity. Despite a low risk injury and technically appropriate surgery, this cellular defect was associated with a delayed union that required additional surgery.

Although other studies have examined the molecular basis of nonunions in vitro, they have done so on heterogeneous populations of bone marrow stromal cells, isolated from established nonunions.^(^ ^8^, ^10^ ^)^ Plastic adherent cells expressed surface markers consistent with a bone marrow stromal cell phenotype, or with cellular senescence.^(^ ^9^ ^)^ Furthermore, previous studies focused on established nonunions, where the cellular defect that led to nonunion pathogenesis probably occurred early in the fracture healing period. By contrast, this report focuses on primary SSCs from acute fractures. hSSCs can be reliably isolated on the basis of cell surface markers and represent the most rigorously validated skeletal stem cell population to date. Strikingly, we also found that other cell types commonly present in plastic‐adherent cultures show divergent osteogenic differentiation capacity.

Despite the fact that human SSCs were abundantly present in both reported cases, the fracture‐derived stem cells of one young healthy individual demonstrated intrinsic osteogenic defects in vitro that correlated with impaired healing. Typically, hSSCs from young individuals demonstrate robust clonogenicity and osteogenesis.^(^ ^18^ ^)^ Although association does not imply causation, these data suggest that an evaluation of hSSCs at time of surgery may potentially predict clinical healing outcome.

Our findings are restricted in generalizability by the intrinsic limitations of a case report: This was a single patient with an adverse healing outcome. We recently reported on a clinical series of 61 patients that prospective SSC isolation from fracture sites showed comparable functional readouts in patients of similar age, even across multiple skeletal sites.^(^ ^20^ ^)^ In this case, the index patient was treated with bridge plating and intended secondary bone healing, whereas the comparison was treated with compression plating. Thus, slightly different surgical techniques were used, although both were viable fixation strategies. Whereas one fracture pattern was a diaphyseal ulna and the other was a diaphyseal radius and ulna pattern, both fractures have high healing rates in young individuals. Furthermore, hSSCs from fractures of young individuals typically are robust in frequency, CFUs‐F (clonogenicity), and osteogenic capacity.^(^ ^18^ ^)^ To identify a prospective cohort of nonunions, several hundred fractures would need to be analyzed, given the rare incidence of nonunions. The CFU‐F percentage and PDPN+CD146 percentage were both increased in patient A. It is possible that clonogenicity decreases in activated SSCs at injury sites that become poised to differentiate along the osteochondrogenic lineage, explaining the decreased CFUs‐F in patient B. Additionally, we have observed SSC abundance does not correlate with CFU‐F percentage, and that functional differentiation capacity correlates more closely with differences in fracture healing than quantitative differences in other subpopulations.^(^ ^20^ ^)^ In this study, the assays performed were limited to in vitro assessments of clonogenicity and skeletal differentiation. However, the in vivo stem cell properties of hSSCs have previously been validated.^(^ ^18^ ^)^ Although it is impossible to precisely compare cell populations isolated by surgical dissection between patients, the prospective flow cytometric analysis and the limitation to fracture hematoma ensure that the study populations are as similar as possible.^(^ ^20^ ^)^ The in vitro assays take 14 to 21 days after isolation to finalize, which constrains their prognostic capability as clinical tests per se, but could, in the future, be correlated with more rapid, sensitive assays such as differential gene expression.

These data provide proof of principle that an assessment of an individual's risk of compromised fracture healing can be made at time of fracture fixation surgery using functional analysis of prospectively isolated hSSCs. In the future, this risk assessment could lead to improved prognostication, as well as additional interventions, to optimize fracture healing in higher‐risk patients. Further studies are warranted to evaluate the role of hSSC assessment in predicting compromised fracture healing, and to determine the molecular mechanism by which hSSCs contribute to normal and impaired fracture healing.

## Disclosures

Authors report no conflicts of interests.

## AUTHOR CONTRIBUTIONS

**Lawrence Goodnough:** Conceptualization; data curation; formal analysis; investigation; methodology; project administration; resources; software; supervision; validation; visualization; writing‐original draft; writing‐review and editing. **Thomas Ambrosi:** Formal analysis; investigation; methodology; resources; validation; visualization; writing‐original draft; writing‐review and editing. **Holly Steininger:** Data curation; formal analysis; investigation; methodology; writing‐review and editing. **Malcolm DeBaun:** Investigation; methodology; supervision; validation; visualization; writing‐review and editing. **Geoff Abrams:** Resources; supervision; validation; visualization; writing‐review and editing. **Timothy McAdams:** Project administration; validation; visualization; writing‐review and editing. **Michael Gardner:** Data curation; formal analysis; supervision; validation; visualization; writing‐original draft; writing‐review and editing. **Charles Chan:** Conceptualization; data curation; formal analysis; funding acquisition; investigation; methodology; supervision; validation; visualization; writing‐original draft; writing‐review and editing. **Julius Bishop:** Conceptualization; data curation; formal analysis; investigation; methodology; project administration; supervision; validation; visualization; writing‐original draft; writing‐review and editing.

## Data Availability

The peer review history for this article is available at https://publons.com/publon/10.1002/jbm4.10398.
